# Aflatoxin M1 Contamination in Dairy Milk in Kathmandu, Nepal

**DOI:** 10.3390/toxins16110468

**Published:** 2024-11-01

**Authors:** Sujan Kafle, Madhav Paudel, Chanda Shrestha, Khadak Bahadur Kathayat, Ram Chandra Sapkota, Ananda Tiwari, Deepak Subedi

**Affiliations:** 1 Faculty of Animal Science, Veterinary Science and Fisheries, Agriculture and Forestry University (AFU), Chitwan 44200, Nepal; vet.madhav@gmail.com (M.P.); kathayatkhadak407@gmail.com (K.B.K.); 2 Department of Diagnostic Medicine/Pathobiology (DMP), College of Veterinary Medicine, Kansas State University, Manhattan, KS 66502, USA; 3 Central Veterinary Laboratory, Kathmandu 44600, Nepal; chandavetshrestha@gmail.com (C.S.); visitrc2000@gmail.com (R.C.S.); 4 Department of Food Hygiene and Environmental Health, Faculty of Veterinary Medicine, University of Helsinki, 00014 Helsinki, Finland; 5 Department of Poultry Science, University of Georgia, Athens, GA 30602, USA; subedideepu26@gmail.com

**Keywords:** aflatoxin M1, mycotoxin, contamination, milk, food safety, Nepal, Kathmandu

## Abstract

Aflatoxins (AFs), secondary metabolites produced by fungi, pose significant health risks, especially to children and elderly individuals. In developing countries such as Nepal, the tropical climate promotes fungal growth, leading to elevated levels of AF in animal feed and milk. In this study, we aimed to investigate the occurrence of aflatoxin M1 (AFM1) in dairy milk from the Kathmandu District and to assess husbandry practices contributing to contamination. We collected 84 milk samples, including raw milk from farms, retailers’ milk, and packet milk, and analyzed them using the competitive enzyme-linked immunosorbent assay (c-ELISA) technique. We also interviewed farmers to gather information on feeding and storage practices. All the collected milk samples were contaminated with AFM1, with 97.6% of the samples exceeding the European Union (EU) maximum permissible limit of 50 ppt (0.05 μg/kg). The majority (98.5%) of the farms included paddy straw, and all farms (100%) included concentrate in their feed regimens. Only half (52%) of the farms had proper storage facilities. Straw was mostly stored in sacks outdoors or left open in a shed, while concentrates were stored in a closed room or shed. This study reveals very high levels of AFM1 contamination in the milk samples, presenting a serious public health issue, and recommends comprehensive surveillance and further investigations across the country, especially given the limited research and literature available.

## 1. Introduction

Mycotoxins are naturally occurring, low-molecular-weight toxic chemicals produced as secondary metabolites by certain filamentous fungi [1]. Among over 300 mycotoxins detected and reported, aflatoxins are among the most commonly found and highly toxic and present a significant global food safety concerns [2]. Aflatoxins are primarily produced by *Aspergillus flavus*, *Aspergillus parasiticus*, and, infrequently *Aspergillus nomius*, which grow on various food commodities such as cereals, peanuts, groundnuts, cottonseeds [3]. Among the dozens of identified aflatoxins, aflatoxin B1, B2, G1, G2, and M1 are considered important due to their toxicity and associated health risks to both domesticated animals and humans [2]. Aflatoxins B and G are usually found in dairy animal feeds, which are major sources of exposure of AFM1 in milk [4].

**Figure 1:**
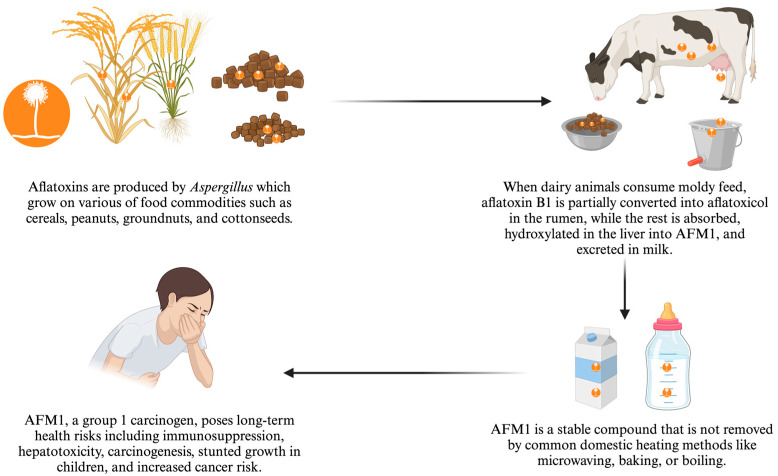
Aflatoxin contamination in milk from feed.

Aflatoxin M1 (AFM1) is a metabolite of aflatoxin B1 that is excreted through the milk, thereby posing a public health risk to milk consumers [5,6]. AFM1 is a stable compound that is not eliminated by common domestic heating methods such as microwaving, baking, or boiling [5]. However, its stability during pasteurization is debated. Some studies suggest that pasteurization does not affect AFM1 levels [7,8,9], while others report a 16% reduction, possibly due to the breakdown of casein during heat treatment [10]. Agricultural residues such as rice straw, wheat straw, maize stovers, and crop hulls, along with feed components such as maize grain, rice bran, and wheat bran, are important animal feeds but are highly susceptible to aflatoxin contamination, particularly under poor storage conditions [11,12]. When dairy animals consume moldy feeds contaminated with aflatoxin, they ingest AFB1, which is partially converted in the rumen into aflatoxicol (Figure 1). The remaining AFB1 is absorbed through the digestive tract, hydroxylated in the liver by cytochrome P450 enzymes into AFM1, and then excreted in milk [13].

Although AFM1 is less toxic than AFB1, it still poses serious long-term health risks, especially to children and elderly individuals, as it is a potent immunosuppressive agent, hepatotoxin, and carcinogen [14]. AFM1, along with AFB1, AFB2, AFG1, and AFG2, is categorized as a Group 1 carcinogen by the International Agency for the Research on Cancer (IARC) [15]. Exposure to AFM1 in early life and childhood is associated with reduced Height-for-Age Z (HAZ) scores in children [16], reduced birth weight [17], reduced height at birth [5], and stunted growth [18]. The cumulative effects of the toxin are also correlated with increased risk of cancer, immune suppression, hepatocellular carcinoma, Reye’s syndrome, cirrhosis, and Kwashiorkor [1,19]. A study in Egypt showed a correlation between increased AFM1 levels in blood and a high hepatitis C virus titer in patients with chronic liver disease [20].

Aflatoxin contamination in human food and dairy products is a global issue, that affects approximately 25% of food products annually, with particularly severe impacts in developing and underdeveloped countries due to poor husbandry practices and socioeconomic conditions [21]. The European Commission has set the maximum permissible level (MPL) for AFM1 as 25 ppt (parts per trillion) for infant formulae and 50 ppt for raw milk [22]. These MPLs are often found to be violated by a marketed milk in many African, Middle East, and South Asian countries [13]. In some studies from these regions, the prevalence of AFM1 in milk exceeding the EU limit has been found to be 100%, highlighting a concerning food safety situation [23].

Nearly all Nepalese individuals, from children to the elderly, consume milk; however, there is a significant lack of information about AFM1 contamination in milk, and the public remains largely unaware of the potential health risks associated with consuming AFM1-contaminated milk [24,25]. This issue is particularly severe in the Kathmandu District, where milk consumption is high, yet milk is rarely screened for adulteration and contamination, leaving its safety for human consumption uncertain. A study by Pokharel et al. (2021) found AFM1 in 94% (i.e., 1355/1439) of breast milk samples, highlighting the significant risk of toxin exposure to humans in Nepal [25]. Similarly, research in neighboring countries such as India, Pakistan, and China has reported that AFM1 levels in milk exceeding permissible limits (≥50 ppt) [23,26,27,28], reflecting broader regional concerns.

This study aimed to determine the occurrence of AFM1 in various types of milk—raw farm, dairy retailers’, and packet milk—in the Kathmandu District and to compare the contamination levels with international standards to evaluate the safety of milk for human consumption. Additionally, the study sought to assess different husbandry practices that contribute to mold infestation and aflatoxin contamination, providing guidance to relevant authorities in developing strategic measures to mitigate this issue.

## 2. Results

### 2.1. Aflatoxin Contamination

Milk samples collected from eight municipalities in the Kathmandu District were analyzed for aflatoxin M1 using a competitive enzyme-linked immunosorbent assay (c-ELISA). All 84 tested milk samples had aflatoxin levels above the lower limit of detection (5 ppt), indicating that 100% of the milk samples were contaminate (Table 1). Of these, 82 samples (97.6%) exceeded the EU’s maximum permissible limit of 50 ppt. The two samples that did not exceed the EU limit were from pooled milk collected from farms.

### 2.2. Farm Characteristics

Forty-eight cattle farms from the Kathmandu District were enrolled in the study. Among the farmers who responded to the questionnaire, the average age was 39 years, and 70.8% were male. Only two respondents had ever heard of aflatoxin contamination in milk. Most of the respondents were small-scale herders, with an average herd size of 15.8 animals per farm (SD = 17.3), ranging from 4 to 86 animals (Q1 = 6, Q2 = 8, and Q3 = 20). Daily milk yield varied from 9 L to 400 L, with an average of 88.8 L. Of these farms, 87.5% were managed intensively, while the remaining 12.5% were managed semi-intensively with mixed grazing practices. The two farms with AFM1 levels below 50 ppt both had smaller herd sizes and were located in pre-urban areas (Table 2). 

### 2.3. Feeding Practice

All farms, except for two, included cut-and-carry forage in their livestock feeding practices. The exceptions relied on other feed sources such as concentrates and straw. Only three farms incorporated silage into their feeding regimens. On 98.5% of the farms, the feedstuffs consisted of paddy straw, with all but one sourced from the Terai, the southern region of the country. All farms used concentrates, either as “homemade, ready-made feed” or “commercial compound pellet feed.” Eleven farms exclusively used commercial feed, two relied solely on homemade feed, and the remaining 35 farms used a combination of both in varying ratios. Twenty-three farms also included unconventional feedstuffs, such as leftovers and brewers’ dry yeast. Crushed maize and paddy husk were the primary ingredients in the “homemade ready-made” feed. The inclusion percentages of the various ingredients in “homemade ready-made feed” and the different unusual feedstuffs are provided in the Supplementary Files (Tables S1 and S2). None of the farms used antitoxins in their regimens. Data on the inclusion of these ingredients in animal feed and the percentage of samples exceeding the EU limit are presented in (Table 3). 

### 2.4. Storage Practice

**Figure 2:**
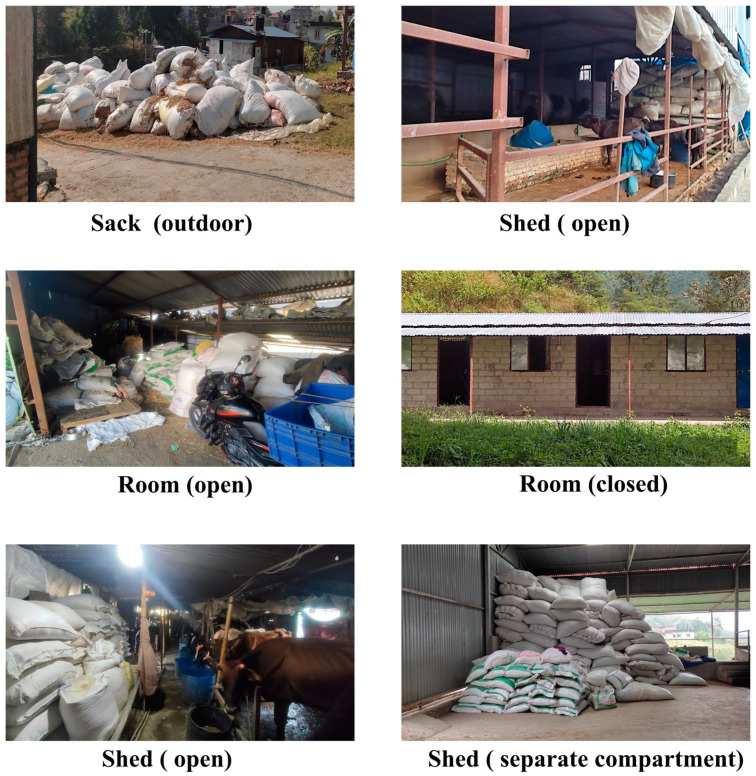
Different storage methods for storing straw and concentrates in dairy farms of the Kathmandu District, Nepal.

Although farmers were aware of mold infestations in stored feedstuffs, they had few options other than to store them for future use, particularly for dry fodder (straw) sourced from outside the valley, especially the Terai region of the country. The duration of straw storage (mean = 35.76 days, SD = 34 days, IQR = 19–30 days) was longer than that of the concentrates (mean = 22.40 days, SD = 10.7 days, IQR = 15–30 days). Farmers stored straw for up to 6 months and concentrates for up to 2 months. Only 52% of farms had dedicated storage facilities, most of which were simply open spaces allocated for storage next to the farms. Farmers used different storage methods for storing straw and concentrates, including sacks, sheds (open or with compartments), and rooms (with open or closed doors), as illustrated in (Figure 2), and adopted various storage practices (Table 4). 

## 3. Discussion

This study was conducted to assess AFM1 contamination in milk samples from the Kathmandu District. Given the limited data and scarce research on AFM1 in milk in Nepal, this study aimed to provide insights into the current situation and guide future surveillance in the region and other parts of the country. The results were alarmingly concerning. Among the 84 milk samples analyzed by competitive ELISA for AFM1, all samples exhibited some level of contamination, indicating a 100% occurrence of AFM1 in the milk. Even more troubling, 97.6% of the samples had AFM1 levels exceeding the EU’s permissible limit of 50 ppt, a standard set for human consumption. This presents a serious food safety issue for consumers in the Kathmandu District. Because the same milk is used to produce dairy products such as yogurt, cheese, and sweets, these products may also exhibit comparable contamination trends similar to those reported in India and Pakistan [28,29].

Several studies from South Asian countries have reported very high levels of AFM1 contamination in milk and milk products, and our study is no exception. The prevalence rate in Nepal is also comparable to findings from the Middle East, Africa, and South America [23]. For example, Iran has documented a significant number of research articles on AFM1, highlighting it as a serious issue, with multiple reports indicating a 100% prevalence rate [30]. Similar findings have been reported in Ethiopia, where Tadesse et al. (2017) and Gizachew et al. (2016) reported AFM1 contamination in 100% of milk samples [31,32]. In Kenya and South Africa also, studies reported occurrence levels above 85% [33,34]. In Brazil, AFM1 was found in 100% of pasteurized and ultra heat-treated (UHT) milk samples [35]. Contamination levels above 90% have also been reported in Thailand [36], Taiwan [37], and Indonesia [38]. This widespread aflatoxin contamination in milk and dairy products in tropical countries is largely attributed to the common contamination of crops, crop residues, and animal feedstuffs [39]. The environmental conditions in tropical areas are conducive to fungal growth and mycotoxin production [40]. The problem is particularly severe in low- and middle-income countries, where poor harvesting, storage, and transportation practices are prevalent, and where policies or the implementation of regulations and mitigation strategies are often lacking [41].

Aflatoxin M1 contamination in milk is associated with various risk factors, with the primary source being the feedstuffs consumed by the animals [42]. Therefore, the feed regimen and storage practices are critical factors associated with AFM1 contamination in milk. All farms in the study relied on concentrates, with most using a mixture of homemade ready-made feed and commercial pellet feed, both of which have been frequently recognized as potential risk factors for AFM1 contamination in milk [43,44]. Although evaluating aflatoxin levels in cattle feed in Nepal is crucial given the sparse literature on the subject—this aspect was not addressed in our study. However, a study on poultry feed by Aryal and Karki (2009) revealed a high prevalence (75.4%) of aflatoxin B1 and B2, with levels as high as 366 µg/kg [45].

The primary ingredients in both the commercially produced and homemade concentrates in our study—crushed maize, paddy husk, mustard oil cake, and wheat bran—are particularly susceptible to mold infestation and mycotoxin contamination, especially during storage [27]. Joshi et al. (2022) reported that 78% of maize samples were contaminated with aflatoxin, with a mean concentration of 23.04 ppb, exceeding the permissible limit (20 ppb) established by the government of Nepal [46]. Other studies have also reported the contamination of maize with aflatoxin in Nepal [46,47,48]. In our study, all farms relying on commercial pellet feed had AFM1 levels above the EU limits and exhibited higher contamination levels. Studies by Kang’ethe and Lang’a (2009) and Akbar et al. (2020) found a positive correlation between higher aflatoxin contamination in commercial concentrate feed and increased AFM1 concentrations in milk [11,49]. The possible reasons for this include the poor quality of raw materials, suboptimal harvesting practices, and inadequate storage and shipping procedures for both raw materials and the final product. Additionally, 53% of farms supplemented animal diets with unconventional feedstuffs, mainly leftover grains, vegetables, and fruits, likely due to the scarcity of fodder and roughage and the high cost of concentrates. Patyal et al. (2021) found that the inclusion of leftover grains was significantly associated with higher AFM1 levels in milk [43].

Storage facilities and the duration of feed storage, particularly for concentrates, have been found to be significantly associated with aflatoxin contamination in animal feed and milk [43,44]. Half of the farms in our study lacked proper storage facilities; roughages and concentrates were stored in open areas, either in sheds or outdoors. Observations during farm visits revealed that farms in urban areas had limited space to extend sheds for additional storage, leading to the shared use of sheds for storing straw and concentrates in sacks. In contrast, farms on the outskirts, being more spacious, often had designated storage areas, although these were not always equipped with proper facilities. Among all the samples tested, only two did not exceed the EU MPL, both of which were from peri-urban farms. The lower level of AFM1 in peri-urban farms, as compared to urban farms, could be due to the presence of spacious and designated storage areas that help prevent mold growth. Paddy straw, a byproduct of seasonal rice cultivation, constituted a major component of the animals’ diet and was usually procured in bulk mainly from the southern part of the country. Straw was stored for an average of 35 days, with some farms storing it for up to 6 months. Inadequate transportation and improper storage practices promote fungal growth and mycotoxin production in these roughages [50].

This study focused exclusively on milk produced within the Kathmandu District, selecting only farms and dairy retailers that source their milk from this area. However, the milk produced by these farms significantly falls short of meeting the demand in Kathmandu, the country’s most populous city and capital. To address this shortfall, milk is imported from neighboring districts, particularly the Kavre District, which also needs to be assessed to determine the AFM1 contamination level in Kathmandu. The AFM1 concentration could vary in the milk from this region because of different feeding and storage practices on farms situated in areas with abundant forages and ample storage space, unlike Kathmandu.

In conclusion, this study has revealed an alarming 100% occurrence of AFM1 contamination in milk samples from the Kathmandu District, with 97.6% of the samples exceeding the EU’s maximum permissible level. These findings highlight a significant food safety issue, particularly concerning children, who are encouraged to consume milk daily as a nutritional staple; therefore, urgent action is needed to address this critical concern. Similarly, the widespread AFM1 contamination of milk reflects the pervasive mold infestation and aflatoxin production in animal feedstuffs, which can occur at any stage—from harvest to transportation, production, and storage. Each of these stages is vulnerable to mold infestation when practices are inadequate or improper. For example, delayed and slow post-harvest drying in hot, humid monsoon weather, as well as storing grains or feeds on earthen floors or outdoors, promotes mold growth [51]. These are critical points where control strategies could be targeted. For example, encouraging farmers to adopt improved, inexpensive technologies, such as the use of tarps instead of drying harvests on uncovered grounds, could significantly reduce the risk of mold infestation [52]. Very few governmental and international collaborative projects have been undertaken in Nepal regarding mycotoxin surveillance; however, these efforts have predominantly focused on human foods and cereals, neglecting animal feed. Even within human foods, milk—a staple in Nepal—has been entirely overlooked [53]. There is an urgent need for comprehensive national investigations to establish contamination trends, identify risk factors, and assess the health and economic impacts of aflatoxicosis. Routine and periodic laboratory testing, especially in high dairy production regions and at milk collection centers before processing, should be conducted to monitor contamination levels. Additionally, strengthening the regulations for aflatoxin control in cattle feed, along with mandatory certification for feed manufacturers, will help ensure compliance with safety standards. Furthermore, public education efforts should target dairy farmers, retailers, processing companies, and consumers to increase awareness of the risks and economic consequences of aflatoxin in milk.

## 4. Materials and Methods

### 4.1. Study Design and Study Area

**Figure 3:**
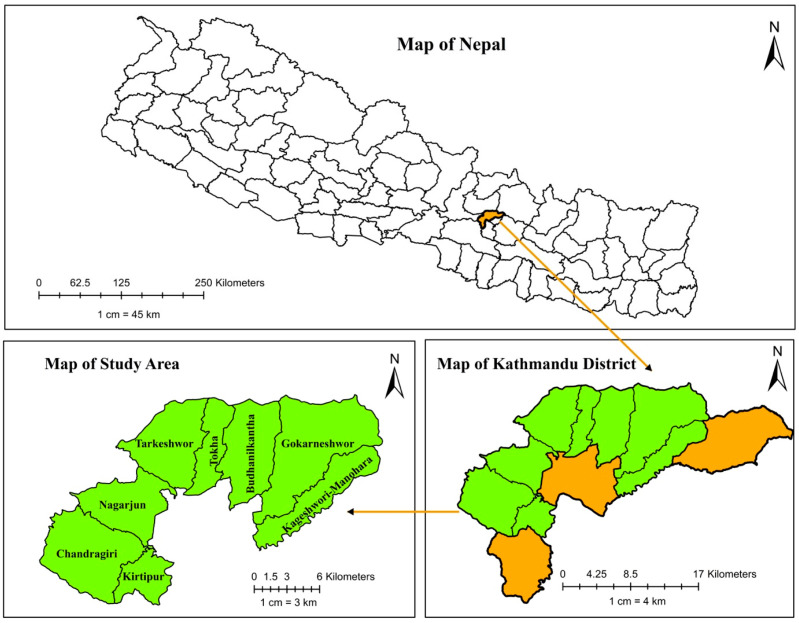
Maps of Nepal and the Kathmandu District showing the study area. Districts highlighted in green indicate the study area.

This cross-sectional study was conducted from October to November 2022 in the Kathmandu District, which is located in the Bagmati Province of central Nepal. The study focused on major milk-producing and distributing areas within the district. Out of the 11 municipalities, eight were selected: Kageswori–Manohara, Kirtipur, Gokarneshwor, Chandragiri, Tokha, Tarkeshwor, Nagarjun, and Budanilakantha (Figure 3). Kathmandu (latitude 27°41′38.76″ N, longitude 85°22′39.00″ E), the nation’s capital, is densely populated and has one of the highest rates of milk and dairy product consumption. The areas within the district were classified as urban and peri-urban; ‘peri-urban’ refers to areas transitioning toward urbanization, whereas “urban” refers to areas that have fully transitioned and exhibit the characteristics of a developed city or town.

### 4.2. Sample Collection

A total of 84 samples were collected for the study and categorized into three groups: farm raw milk (48 samples), dairy retail shop milk (25 samples), and packet milk (11 samples). Forty-eight commercially registered farms with varying herd sizes were randomly selected from eight municipalities within the Kathmandu District. Fresh raw milk samples were collected in sterile, screw-capped 100 mL centrifuge tubes from the bulk milk tanks after proper mixing during early morning visits. Similarly, milk samples were obtained from the bulk tanks of 25 dairy retail shops in these municipalities. These retail shops, which pool milk from various nearby farms, are major distributors of milk in Kathmandu alongside packet milk. Eleven milk packets from different brands were purchased from supermarkets for this study. After collection, all milk samples were placed in an insulated cold box and transported to the Central Veterinary Laboratory, Kathmandu, where they were stored in a refrigerator at −20 °C. All samples were analyzed for AFM1 within two days of collection. During the farm visits for sample collection, a pretested questionnaire was administered via the Kobo Toolbox [54] to farmers, owners, or managers to collect data on farm characteristics, as well as feeding and storage practices. Concentrates refer to low-fiber, high-energy diets made from cereals such as maize, sorghum, paddy, and their byproducts [55]. In Nepal, these concentrates are either sold commercially by animal feed companies in pellet form, packaged in bags—hereafter referred to in the manuscript as ‘commercial compound pellet feed’—or they are prepared by the farmers themselves by grinding and mixing cereals and their byproducts to produce what is hereafter referred to as ‘homemade ready-made feed’ for use in animal rations.

### 4.3. Laboratory Analysis

The milk samples were thawed and then centrifuged at 1000 rpm for 5 min. The upper layer of fat was removed, and the AFM1 was screened in the defatted milk samples using a competitive ELISA (CUSABIO Technology LLC, Wuhan, China), following the manufacturer’s instructions provided with the ELISA kit. The kit included a precoated ELISA plate, standards (0, 5, 15, 45, and 135 ppt), HRP-conjugate, substrates, wash buffer, and stop solution. Briefly, 100 microliters of standards and milk samples were added to the precoated ELISA plate and incubated for 30 min at 25 °C. The plate was then washed five times, and 100 microliters of HRP conjugate were added to each well, followed by a 15-min incubation at 25 °C. After another five washes, 50 microliters of substrate A and substrate B were added, mixed, and incubated for 15 min at 25 °C in the dark. Finally, 50 microliters of stop solution were added, and the absorbance was measured at 450 nm using an ELISA reader (Multiskan™ FC Microplate Photometer, Thermo Fisher Scientific, Waltham, MA, USA), equipped with Skanlt™ version 6.1 software. The calibration curve was constructed from the standard solutions and their absorbance, and the regression equation (R^2^ = 0.98) was used to quantify the aflatoxin levels.

### 4.4. Statistical Analysis

All data from the questionnaire and laboratory analysis were compiled into an Excel file (Microsoft Excel 2019) and subsequently imported into SPSS version 25 for a descriptive assessment of the farms and husbandry practices. The contamination levels of AFM1 in milk were categorized on the basis of the EU’s maximum permissible limit (MPL) of 50 ppt, above which milk is considered unsafe for human consumption. Consequently, contamination levels were reported as either below or above 50 ppt, indicating whether the milk complied with or exceeded the EU limit.

## Figures and Tables

**Table 1:** Comparison of AFM1 in milk collected from the Kathmandu District, Nepal, by region and sample categories.

Sample	Sample Size	Samples Exceeding EU Limit-AFM1 (%)
**Municipalities**		
Budanilkantha	12	100
Kirtipur	15	100
Tokha	13	77.8
Tarkeswor	6	100
Gokernswor	7	100
Kageswori-manohara	6	100
Chandragiri	7	100
Nagarjun	7	100
**Sample categories**		
Farms’ raw milk	48	95.8
Dairy retailers’ milk	25	100
Packet milk	11	100

**Table 2:** Comparison of AFM1 contamination levels with farm characteristics in dairy farms in the Kathmandu District, Nepal.

Farms Characteristics	Number (%)	Samples Exceeding EU Limit-AFM1 (%)
**A. Location**		
Urban	33 (68.8%)	100%
Pre-Urban	15 (31.3%)	86.7%
**B. Rearing Practice**		
Intensive	42 (87.5%)	97.6%
Semi-Intensive	6 (12.5%)	83.3%
**C. Herd Size**		
0–10	29 (60.4%)	93.1%
11–20	10 (20.8%)	100%
Above 20	9 (18.8%)	100%

**Table 3:** Comparison of AFM1 contamination levels with inclusion percentages of various feedstuffs in animal rations on dairy farms in the Kathmandu District, Nepal.

Inclusion in Animal Feed Diet	Response	Number (%)	Samples Exceeding EU Limit-AFM1 (%)
Cut and carry forage	Yes	46 (95.8%)	95.7
	No	2 (4.2%)	100
Silage	Yes	3 (6.3%)	100
	No	45 (93.8%)	95.6
Concentrates	Yes	48 (100%)	95.8%
	No	0 (0%)	
Inclusion of unusual feedstuffs in animal diet	Yes	23 (47.9%)	100%
	No	25 (52.1%)	92.0%
Types of concentrate in animal diet	Only commercial pellet	11 (22.9%)	100%
	Only homemade concentrate	2 (4.2%)	50%
	Mixtures of both	35 (72.9%)	97.1%

**Table 4:** Comparison of AFM1 contamination levels with various storage practices of animal feed on dairy farms in the Kathmandu District, Nepal.

Storage Practice of Animal Feed		Frequency (%)	Samples Exceeding EU Limit-AFM1 (%)
**Storage of dry fodder (straw)**	Sack (outdoor)	18 (40%)	100%
	Shed (open)	16 (35.6%)	93.8%
	Shed (separate compartment)	3 (6.7%)	100%
	Room	6 (13.3%)	100%
	Others	2 (4.4%)	100%
Storage of concentrates	Room (closed)	24 (50%)	91.7%
	Room (Open)	7 (14.6%)	100%
	Shed (open and/or in compartment)	15 (31.3%)	100%
	others	2 (4.2%)	100%
Floor of storage facilities	Raised	24 (50%)	100%
	Floored (Unraised)	24 (50%)	91.7%
Regular monitoring of temperature, humidity and moisture in storage facilities	Yes	0 (0%)	
	No	48 (100%)	95.8%
Have you seen mold infestation in storage facilities?	Yes	13 (27.1%)	100%
	No	35 (72.9%)	94.3%
Have you seen rodent and insect infestation in storage facilities?	Yes	33 (68.8%)	100%
	No	15 (31.3%)	86.7%

## Data Availability

Data are included in the article and the Supplementary Files. Further inquiries or data requests can be directed to the authors.

## Supplementary Materials

The following supporting information can be downloaded at: https://www.mdpi.com/article/10.3390/toxins16110468/s1, Table S1: Inclusion percentage of various ingredients in ‘homemade ready-made feed’ in dairy farms in Kathmandu District, Nepal; Table S2: Inclusion percentage of different unusual feedstuffs in dairy farms in Kathmandu District, Nepal.

## References

[1] Bennett J.W., Klich M. (2003). Mycotoxins. Clin. Microbiol. Rev..

[2] Zain M.E. (2011). Impact of Mycotoxins on Humans and Animals. J. Saudi Chem. Soc..

[3] Alshannaq A., Yu J.-H. (2017). Occurrence, Toxicity, and Analysis of Major Mycotoxins in Food. Int. J. Environ. Res. Public Health.

[4] Kumar A., Pathak H., Bhadauria S., Sudan J. (2021). Aflatoxin Contamination in Food Crops: Causes, Detection, and Management: A Review. Food Prod Process Nutr..

[5] Giovati L., Magliani W., Ciociola T., Santinoli C., Conti S., Polonelli L. (2015). AFM_1_ in Milk: Physical, Biological, and Prophylactic Methods to Mitigate Contamination. Toxins.

[6] Esam R.M., Hafez R.S., Khafaga N.I.M., Fahim K.M., Ibrahim Ahmed L. (2022). Assessment of Aflatoxin M1 and B1 in Some Dairy Products with Referring to the Analytical Performances of Enzyme-Linked Immunosorbent Assay in Comparison to High-Performance Liquid Chromatography. Vet. World.

[7] Al-Delaimy K., Mahmoud I. (2015). Aflatoxin M1 in Milk and Milk Products in Jordan and Methods for Its Reduction: A Preliminary Study. BJAST.

[8] Iha M.H., Barbosa C.B., Okada I.A., Trucksess M.W. (2011). Occurrence of Aflatoxin M1 in Dairy Products in Brazil. Food Control.

[9] Omeiza G.K., Mwanza M., Enem S.I., Godwin E., Adeiza M.A., Okoli C. (2018). Reducing Efficiencies of the Commonly Used Heat Treatment Methods and Fermentation Processes on Aflatoxin M1 in Naturally Contaminated Fresh Cow Milk. OJVM.

[10] Deveci O., Sezgin E. (2006). Changes in Concentration of Aflatoxin M1 during Manufacture and Storage of Skim Milk Powder. J. Food Prot..

[11] Kang’ethe E.K., Lang’a K.A. (2009). Aflatoxin B1 and M1 Contamination of Animal Feeds and Milk from Urban Centers in Kenya. Afr. Health Sci..

[12] Sharma H., Jadhav V.J., Garg S.R. (2020). Aflatoxin M1 in Milk in Hisar City, Haryana, India and Risk Assessment. Food Addit. Contam. Part B.

[13] Ismail A., Akhtar S., Levin R.E., Ismail T., Riaz M., Amir M. (2016). Aflatoxin M1: Prevalence and Decontamination Strategies in Milk and Milk Products. Crit. Rev. Microbiol..

[14] Ferrari L., Rizzi N., Grandi E., Clerici E., Tirloni E., Stella S., Bernardi C.E.M., Pinotti L. (2023). Compliance between Food and Feed Safety: Eight-Year Survey (2013–2021) of Aflatoxin M1 in Raw Milk and Aflatoxin B1 in Feed in Northern Italy. Toxins.

[15] Paniel N., Radoi A., Marty J.-L. (2010). Development of an Electrochemical Biosensor for the Detection of Aflatoxin M1 in Milk. Sensors.

[16] Kiarie G.M., Dominguez-Salas P., Kang’ethe S.K., Grace D., Lindahl J. (2016). Aflatoxin Exposure among Young Children in Urban Low-Income Areas of Nairobi and Association with Child Growth. AJFAND.

[17] Abdulrazzaq Y.M., Osman N., Yousif Z.M., Al-Falahi S. (2003). Aflatoxin M_1_ in Breast-Milk of UAE Women. Ann. Trop. Paediatr..

[18] Mahdavi R., Nikniaz L., Arefhosseini S.R., Vahed Jabbari M. (2010). Determination of Aflatoxin M1 in Breast Milk Samples in Tabriz–Iran. Matern. Child Health J..

[19] Ahlberg S., Grace D., Kiarie G., Kirino Y., Lindahl J. (2018). A Risk Assessment of Aflatoxin M1 Exposure in Low and Mid-Income Dairy Consumers in Kenya. Toxins.

[20] Shahat A., MA S., Mohamed A.F., Abdel-Wahhab P.M. (2012). Correlation Study Between Aflatoxin M 1 and Hepatitis C Virus in Egyptian Patients with Chronic Liver Disease. World J. Med. Sci..

[21] Jallow A., Xie H., Tang X., Qi Z., Li P. (2021). Worldwide Aflatoxin Contamination of Agricultural Products and Foods: From Occurrence to Control. Comp. Rev. Food Sci. Food Safe.

[22] Torović L. (2015). Aflatoxin M _1_ in Processed Milk and Infant Formulae and Corresponding Exposure of Adult Population in Serbia in 2013–2014. Food Addit. Contam. Part B.

[23] Mollayusefian I., Ranaei V., Pilevar Z., Cabral-Pinto M.M.S., Rostami A., Nematolahi A., Khedher K.M., Thai V.N., Fakhri Y., Mousavi Khaneghah A. (2021). The Concentration of Aflatoxin M1 in Raw and Pasteurized Milk: A Worldwide Systematic Review and Meta-Analysis. Trends Food Sci. Technol..

[24] Chamlagain S., Dahal T. (2020). Status of Production and Distribution of Fresh Milk by Villagers in Bariyarpatti, Sohpur. J. Mgt..

[25] Pokharel A., Webb P., Andrews-Trevino J., Lamichhane A., Shrestha R., Acharya S., Davis D., Baral K., Wang J.-S., Xue K. (2021). Prevalence and Associated Factors of Breastmilk Aflatoxin M1 Levels in Mothers from Banke, Nepal. Food Control.

[26] Asghar M.A., Ahmed A., Asghar M.A. (2018). Aflatoxin M _1_ in Fresh Milk Collected from Local Markets of Karachi, Pakistan. Food Addit. Contam. Part B.

[27] Dhavan A.S., Choudary M.R. (1995). Incidence of Aflatoxins in Animal Feedstuffs: A Decade’s Scenario in India. J. Aoac Int..

[28] Hattimare D., Shakya S., Patyal A., Chandrakar C., Kumar A. (2022). Occurrence and Exposure Assessment of Aflatoxin M1 in Milk and Milk Products in India. J. Food Sci. Technol..

[29] Iqbal S.Z., Asi M.R., Jinap S. (2013). Variation of Aflatoxin M1 Contamination in Milk and Milk Products Collected during Winter and Summer Seasons. Food Control.

[30] Rahmani J., Alipour S., Miri A., Fakhri Y., Riahi S.-M., Keramati H., Moradi M., Amanidaz N., Pouya R.H., Bahmani Z. (2018). The Prevalence of Aflatoxin M1 in Milk of Middle East Region: A Systematic Review, Meta-Analysis and Probabilistic Health Risk Assessment. Food Chem. Toxicol..

[31] Tadesse B.T., Ashley E.A., Ongarello S., Havumaki J., Wijegoonewardena M., González I.J., Dittrich S. (2017). Antimicrobial Resistance in Africa: A Systematic Review. BMC Infect. Dis..

[32] Gizachew D., Szonyi B., Tegegne A., Hanson J., Grace D. (2016). Aflatoxin Contamination of Milk and Dairy Feeds in the Greater Addis Ababa Milk Shed, Ethiopia. Food Control.

[33] Kuboka M.M., Imungi J.K., Njue L., Mutua F., Grace D., Lindahl J.F. (2019). Occurrence of Aflatoxin M1 in Raw Milk Traded in Peri-Urban Nairobi, and the Effect of Boiling and Fermentation. Infect. Ecol. Epidemiol..

[34] Mulunda M., Mike D. (2014). Occurrence of Aflatoxin M1 from Rural Subsistence and Commercial Farms from Selected Areas of South Africa. Food Control.

[35] Sifuentes dos Santos J., França V., Katto S., Santana E.H. (2015). Aflatoxin M1 in Pasteurized, UHT Milk and Milk Powder Commercialized in Londrina, Brazil and Estimation of Exposure. Arch. Latinoam. Nutr..

[36] Ruangwises S., Saipan P., Ruangwises N., Razzaghi-Abyaneh M. (2013). Occurrence of Aflatoxin M1 in Raw and Pasteurized Goat Milk in Thailand. Aflatoxins—Recent Advances and Future Prospects.

[37] Lin L.-C., Liu F.-M., Fu Y.-M., Shih D.Y.-C. (2020). Survey of Aflatoxin M1 Contamination of Dairy Products in Taiwan. J. Food Drug Anal..

[38] Sumantri I., Purwanti F., Nuryono N., Agus A. (2019). Estimation of Aflatoxin M1 Exposure through Consumption of Various Dairy Milk Products in Yogyakarta, Indonesia (Estimasi Paparan Aflatoksin M1 Melalui Konsumsi Berbagai Produk Susu Di Yogyakarta, Indonesia). J. Vet..

[39] Assaf J.C., Nahle S., Chokr A., Louka N., Atoui A., El Khoury A. (2019). Assorted Methods for Decontamination of Aflatoxin M1 in Milk Using Microbial Adsorbents. Toxins.

[40] Ashiq S. (2015). Natural Occurrence of Mycotoxins in Food and Feed: Pakistan Perspective. Comp. Rev. Food Sci. Food Safe.

[41] Mamo F.T., Abate B.A., Tesfaye K., Nie C., Wang G., Liu Y. (2020). Mycotoxins in Ethiopia: A Review on Prevalence, Economic and Health Impacts. Toxins.

[42] Yunus A.W., Ullah A., Lindahl J.F., Anwar Z., Ullah A., Saif S., Ali M., Zahur A.B., Irshad H., Javaid S. (2020). Aflatoxin Contamination of Milk Produced in Peri-Urban Farms of Pakistan: Prevalence and Contributory Factors. Front. Microbiol..

[43] Patyal A., Gill J.P.S., Bedi J.S., Aulakh R.S. (2021). Assessment of Aflatoxin Contamination in Dairy Animal Concentrate Feed from Punjab, India. Environ. Sci. Pollut. Res..

[44] Tadele F., Demissie B., Amsalu A., Demelash H., Mengist Z., Ambelu A., Yenew C. (2023). Aflatoxin Contamination of Animal Feeds and Its Predictors among Dairy Farms in Northwest Ethiopia: One Health Approach Implications. Front. Vet. Sci..

[45] Aryal S.R., Karki D. (2009). Prevalence of Aflatoxin B1 and B2 in Poultary Feed. Nepal Agric. Res. J..

[46] Joshi P., Chauysrinule C., Mahakarnchanakul W., Maneeboon T. (2022). Multi-Mycotoxin Contamination, Mold Incidence and Risk Assessment of Aflatoxin in Maize Kernels Originating from Nepal. Microbiol. Res..

[47] Gautam D.N., Bhatta R., Bhandary M.R. (2008). Assessment of Aflatoxin B1 Level in Chilli, Maize and Groundnut Samples from Kathmandu Valley. J. Food Sci. Technol. Nepal.

[48] Pokhrel P. (2016). Postharvest Handling and Prevalence of Aflatoxin Contamination in Nepalese Maize Produce. J. Food Sci. Technol. Nepal.

[49] Akbar N., Nasir M., Naeem N., Ahmad M., Saeed F., Anjum F.M., Iqbal S., Imran M., Tufail T., Shah F. (2020). Assessment of Aflatoxin in Milk and Feed Samples and Impact of Seasonal Variations in the Punjab, Pakistan. Food Sci. Nutr..

[50] Phillips S.I., Wareing P.W., Dutta A., Panigrahi S., Medlock V. (1996). The Mycoflora and Incidence of Aflatoxin, Zearalenone and Sterigmatocystin in Dairy Feed and Forage Samples from Eastern India and Bangladesh. Mycopathologia.

[51] Neme K., Mohammed A. (2017). Mycotoxin Occurrence in Grains and the Role of Postharvest Management as a Mitigation Strategies. A Review. Food Control.

[52] Adenitan A.A., Awoyale W., Akinwande B.A., Busie M.-D., Michael S. (2021). Mycotoxin Profiles of Solar Tent-Dried and Open Sun-Dried Plantain Chips. Food Control.

[53] PHLIL N. (2020). Feed the Future Innovation Lab for the Reduction of Post-Harvest Loss. PHLIL Nepal Buy-In: Final Report.

[54] KoboToolbox. https://www.kobotoolbox.org/.

[55] Ishler V.A., Becker C. Concentrates for Dairy Cattle. https://extension.psu.edu/concentrates-for-dairy-cattle.

