# Supplementary material for: Aflatoxin M1 Contamination in Dairy Milk in Kathmandu, Nepal
Source: Toxins (Basel). 2024 Nov 1;16(11):468. doi: 10.3390/toxins16110468 (PMC11598514; doi:10.3390/toxins16110468)
Supplement: Supplementary file 1 [file toxins-16-00468-s001.zip › toxins-3223186-supplementary.pdf]

toxins

MDPI

Supplementary materials

Aflatoxin M1 Contamination in Dairy Milk in Kathmandu, Nepal

Sujan Kafle, Madhav Paudel, Chanda Shrestha, Khadak Bahadur Kathayat, Ram Chandra Sapkota, Ananda Tiwari and Deepak Subedi

Table S1. Inclusion percentage of various ingredients in 'homemade ready-made feed' in dairy farms in Kathmandu district, Nepal.

**Feedstuff**	**Inclusion % by farms**
Maize	95%
Paddy husk	95%
Mustard oil cake	85%
Wheat bran	72%

Table S2. Inclusion percentage of different unusual feedstuffs in dairy farms in Kathmandu district, Nepal.

**Unusual Feedstuff**	**Inclusion % as per by farms**
Left over grains	39.6
Leftover vegetables and fruits	39.6
Stale bread	16.7
Brewers dry yeast	6.3
